# Chloroquine and mefloquine resistance profiles are not related to the circumsporozoite protein (CSP) VK210 subtypes in field isolates of *Plasmodium vivax* from Manaus, Brazilian Amazon

**DOI:** 10.1590/0074-02760190054

**Published:** 2019-08-12

**Authors:** Lilian Rose Pratt-Riccio, Bárbara de Oliveira Baptista, Vanessa Rodrigues Torres, Cesare Bianco-Junior, Daiana de Souza Perce-Da-Silva, Evelyn Kety Pratt Riccio, Josué da Costa Lima-Junior, Paulo Renato Rivas Totino, Gustavo Capatti Cassiano, Luciane Moreno Storti-Melo, Ricardo Luiz Dantas Machado, Joseli de Oliveira-Ferreira, Dalma Maria Banic, Leonardo José de Moura Carvalho, Cláudio Tadeu Daniel-Ribeiro

**Affiliations:** 1 Fundação Oswaldo Cruz, Instituto Oswaldo Cruz, Laboratório de Pesquisa em Malária, Rio de Janeiro, RJ, Brasil; 2 Fundação Oswaldo Cruz, Centro de Pesquisa, Diagnóstico e Treinamento em Malária, Rio de Janeiro, RJ, Brasil; 3 Fundação Oswaldo Cruz, Instituto Oswaldo Cruz, Laboratório de Imunoparasitologia, Rio de Janeiro, RJ, Brasil; 4 Universidade de Campinas, Departamento de Genética, Evolução e Bioagentes, Laboratório de Doenças Tropicais, Campinas, SP, Brasil; 5 Universidade Federal de Sergipe, Centro de Ciências Biológicas e da Saúde, Departamento de Biologia, Aracaju, SE, Brasil; 6 Universidade Federal Fluminense, Instituto Biomédico, Departamento de Microbiologia e Parasitologia, Niterói, RJ, Brasil; 7 Fundação Oswaldo Cruz, Instituto Oswaldo Cruz, Laboratório de Imunologia Clínica, Rio de Janeiro, RJ, Brasil

**Keywords:** malaria, Plasmodium vivax, circumsporozoite protein, chemoresistance

## Abstract

**BACKGROUND:**

The central repetitive region (CRR) of the *Plasmodium vivax* circumsporozoite surface protein (CSP) is composed of a repetitive sequence that is characterised by three variants: VK210, VK247 and *P. vivax*-like. The most important challenge in the treatment of *P. vivax* infection is the possibility of differential response based on the parasite genotype.

**OBJECTIVES:**

To characterise the CSP variants in *P. vivax* isolates from individuals residing in a malaria-endemic region in Brazil and to profile these variants based on sensitivity to chloroquine and mefloquine.

**METHODS:**

The CSP variants were determined by sequencing and the sensitivity of the *P. vivax* isolates to chloroquine and mefloquine was determined by Deli-test.

**FINDINGS:**

Although five different allele sizes were amplified, the sequencing results showed that all of the isolates belonged to the VK210 variant. However, we observed substantial genetic diversity in the CRR, resulting in the identification of 10 different VK210 subtypes. The frequency of isolates that were resistant to chloroquine and mefloquine was 11.8 and 23.8%, respectively. However, we did not observe any difference in the frequency of the resistant isolates belonging to the VK210 subtypes.

**MAIN CONCLUSION:**

The VK210 variant is the most frequently observed in the studied region and there is significant genetic variability in the CRR of the *P. vivax* CSP. Moreover, the antimalarial drug sensitivity profiles of the isolates does not seem to be related to the VK210 subtypes.

Despite remarkable progress in the control of malaria, it still remains a public health problem in several countries where the disease is endemic. According to the latest World Health Organization (WHO) estimates, nearly half of the world’s population is at risk of malaria infection and 91 countries and territories have been classified as endemic. In 2017, 219 million cases and 435,000 deaths from this disease were reported worldwide and the victims were mainly children under the age of five years. Most of these cases (92%) and deaths (93%) occurred in Africa, followed by Southeast Asia and the East Mediterranean Region.^1^ Of the six *Plasmodium* species that infect humans, *Plasmodium vivax* is the most widespread, being responsible for around 3.4% of the estimated global cases. However, outside the African continent, this proportion is over 36%. *P. vivax* is the most predominant parasite in the entire continent of America, representing approximately 74% of all malaria cases. In the East Mediterranean region, it represents over 31% of the cases and in East Asia, 37%.^1^ In Brazil, *P. vivax* represented around 90% of the 194,000 malaria cases registered in 2017.^2^ Although *P. vivax* infection is considered to be clinically milder than *Plasmodium falciparum* infection, there have been cases of severe malaria and death due to *P. vivax* infection in many endemic areas, including Brazil.^3^

The *P. vivax* circumsporozoite surface protein (CSP), which is the most abundant polypeptide present on the surface of the sporozoite, is a well-characterised antigen and one of the few vaccine candidates for*P. vivax*tested in clinical trials.^4^ CSP is involved in the motility and invasion of the sporozoite into the hepatocytes and represents an important vaccine target, since anti-CSP antibodies from naturally infected individuals or from volunteers immunised with irradiated sporozoites are able to inhibit the invasion of hepatocytes by live sporozoites *in vitro.* ^5^ ^,^ ^6^ The *csp* gene encodes for a protein that is characterised by two highly conserved terminal nonrepetitive regions (N- and C-terminal) flanking a highly immunogenic, central repetitive domain. The central repetitive region (CRR) of the CSP is composed of one of two possible nonapeptides that repeats in tandem, GDRA(A/D)GQPA and ANGA(G/D)(N/D)QPG, which are characteristic of theVK210 andVK247 CSP variants, respectively. These nonapeptide sequences are repeated nearly 20 times in their corresponding proteins. Besides these two variants, a third, known as *P. vivax*-like, has an 11-mer repetitive sequence, APGANQ(E/G)GGAA.^7^ ^-^ ^10^ However, there have been reports on polymorphisms related to the number of the residues in these CRR and several synonymous and non-synonymous point mutations.^11^ The CSP variants have been found at variable frequencies in different malaria endemic areas. Previous studies have used serological and molecular analysis to describe the occurrence of these three variants in both pure and mixed infections in Brazil.^6^ ^,^ ^12^ ^,^ ^13^

The distribution of these variants seems to be universal and the infections caused by the CSP variants seems to be associated with vector preference and susceptibility, symptom severity, clinical signs, humoral response patterns, parasite burden and cytokine balance.^10^ ^,^ ^14^ ^-^ ^16^ Another important issue is that the response to the treatment might possibly differ depending on the genotype of the parasite. A study performed by Kain et al.^17^ suggested that the response to chloroquine varies depending on the *P. vivax* CSP variants as both single VK210 as well as VK210/VK247 mixed infections took longer to clear when compared to single VK247 infection in Thailand. 

The first reports of chloroquine-resistant *P. vivax* isolates were obtained from Papua New Guinea and Indonesia in 1989. In Brazil, the first reported case of chloroquine-resistant *P. vivax* was from a patient treated in Manaus, state of Amazonas, in the Brazilian Amazon. Later, subsequent studies assessed the efficiency of standard supervised therapy or the *in vitro* profile of chloroquine-resistance showing failure rates of chloroquine treatment between 5 and 10%^18^ ^,^ ^19^ with approximately 10% chloroquine-resistance profile seen in short-term culture.^20^ *In vitro* resistance of *P. vivax* isolates to mefloquine in Manaus has also been described to be at variable frequencies.^20^

In the present study, we characterised the CSP variants in the *P. vivax* isolates from individuals residing in malaria-endemic area of the Brazilian Amazon and studied the sensitivity profiles of these parasites to chloroquine and mefloquine using short-term *in vitro* cultures. 

## MATERIALS AND METHODS

*Study site and isolates -* This study was carried out in the city of Manaus. A total of 95*P. vivax*isolates were collected from patients who sought health care at Fundação de Medicina Tropical Doutor Heitor Vieira Dourado (FMT-HVD) between 2004 and 2007, as previously described.^20^

We obtained written informed consent from all the donors and venous blood samples were drawn in Vacutainer^®^ (Becton Dickinson, Oxnard, CA, USA) ethylenediamine tetraacetic acid (EDTA) tubes. For determination of drug sensitivity, the tubes containing the blood samples were maintained at 4ºC before the*in vitro*culture was initiated.

For molecular analysis, the tubes were centrifuged at 350 *g* for 10 min to remove the plasma and the pellet was stored at -20ºC. The pellets, containing the peripheral blood cells, were mixed with equal volumes of a cryopreservation solution (0.9% NaCl/4.2% sorbitol/20% glycerol) and were stored in liquid nitrogen until further use. Thin and thick blood smears were examined to identify the malaria parasites and determine presence of parasitaemia by two technicians who were experts in malaria microscopy from FMT-HVD and from the Laboratório de Pesquisa em Malária (Laboratory of Malaria Research) [Fundação Oswaldo Cruz (Fiocruz)], which is the headquarters of the Centro de Pesquisa e Treina-mento em Malária of the Secretaria de Vigilância em Saúde (Center for Malaria Research and Training, Department of Health Surveillance), a reference centre for malaria diagnosis in the extra-Amazonian Region for the Brazilian Ministry of Health. Thick blood smears from all of the subjects were stained with Giemsa and a total of 200 microscopic fields were examined under a 1,000-fold magnification. Thin blood smears of the positive samples were examined for species identification. The parasite density was evaluated by counting the parasites in a predetermined number of white blood cells in the thick blood films, and the number of blood parasites per millilitre was calculated. To increase the sensitivity of the parasite detection, molecular analyses using specific primers for genus (*Plasmodium* sp.) and species (*P. falciparum* and *P. vivax*) were performed for all of the samples. 

All the patients enrolled in this study complied with the following criteria: (i) they presented symptoms; (ii) they were infected with only *P. vivax*; (iii) they did not use any chemoprophylaxis or any antimalarial drugs as self-treatment; (iv) they were 12 years of age or older; (v) women were not pregnant or breast feeding; and, (vi) blood collection was performed on the day of diagnosis before malaria treatment. After the malaria diagnosis and blood sample collection, the patients were immediately treated according to the Brazilian Ministry of Health standards for malaria therapy.

*Ethics statement -* The study protocol was reviewed and approved by the Fiocruz Ethical Committee (protocol 221/03), which included obtaining the patients’ written consents in order to use their blood samples for research. Written informed consent was obtained from all the adult donors or from the parents of the donors in the case of minors. All the procedures adopted in this study fully complied with the specific federal permits issued by the Brazilian Ministry of Health.

*Characterisation of the CSP variants -* The CSP variants were determined by PCR-sequencing. DNA was extracted from the blood samples using the QIAamp DNA blood midi kit (Qiagen, Germantown, MD, USA) according to manufacturer instructions and stored at -20ºC until amplification. The *csp* gene of each sample was amplified by two independent, conventional polymerase chain reaction (PCR) methods using either of the following two pairs of primers: AL60 5’-GTCGGAATTCATGAAGAACTTCATTCTC-3’ (forward) and AL61 5’-CAGCGGATCCTTAATTGAATAATGCTAGG-3’ (reverse), or PVCSP1 5’-AGGCAGAGGACTTGGTGAGA-3’ (forward) and PVCSP2 5’-CCACAGGTTACACTGCATGG-3’ (reverse) (Genone Biotechnologies, Rio de Janeiro, RJ, Brazil). All the PCR amplifications were carried out in a 50 μL reaction mixture containing 8 μL of genomic DNA, 5 μL of 10X PCR buffer (20 mM Tris-HCl pH 8.4, 50 mMKCl), 1.5 mM MgCl_2_, 0.2 mM of each dNTP, 0.2 μM of each primer and 2.5U of Taq polymerase (Invitrogen, California, CA, EUA) kit according to the manufacturer’s. The amplifications were performed in a GeneAmp PCR system 9700 thermal cycler (Applied Biosystem, Foster City, CA, USA) using the following steps: an initial cycle of 94ºC for 10 min followed by 30 cycles of 94ºC for 1 min, 48ºC for 1 min and 72ºC for 1 min, with a final extension at 72ºC for 10 min for the pair of primers AL60/AL61 and an initial cycle of 94ºC for 10 min followed by 30 cycles of 94ºC for 1 min, 60ºC for 1 min and 72ºC for 1 min, with a final extension at 72ºC for 10 min, for the pair of primers PVCSP1/PVCSP2. In all of the reactions, two negative controls (one without DNA and the other with DNA extracted from an *in vitro* culture of *P*. *falciparum* PSS1 strain) and a positive control (*P*. *vivax*-infected sample) were used. Further, 5 μL of the PCR product was electrophoresed at 95V for 90 min along with 0.5 µg/mL 100 base pairs (bp) DNA molecular weight marker (ThermoFisher Scientific, Waltham, MA, USA) in 2% agarose gel (Sigma-Aldrich, St. Louis, MI, USA) in 1x tris-acetate-EDTA (TAE) buffer (0.04 M TAE, 1 mM EDTA), and the gel was stained by ethidium bromide (EtBr). The target DNA was visualised and the images were captured using an ultraviolet transilluminator (Multi-Doc IT Digital Imaging System UVP). The positive samples were electrophoresed in 2% agarose low melting point gel stained with EtBr. Then, the PCR fragments were purified using the Wizard SV Gel and PCR Clean-UP System (Thermo Fisher) kit according to the manufacturer’s protocol and quantified using the Qubit dsDNA HG Assay kit (Invitrogen).

*DNA sequencing and polymorphism analysis -* The specificity of the assay was confirmed by sequencing the PCR products from all of the positive samples using a Big Dye Terminator Ready Reaction version 3.1 (Thermo Fisher), following the manufacturer’s instructions. The products that were amplified with the pair of primers AL60 and AL61 were sequenced with primers AL60, AL61, PVCSP1 and PVCSP2. The PCR products amplified with the primer pair, PVCSP1 and PVCSP2 were sequenced with PVCSP1 and PVCSP2. The DNA sequencing was carried out on the 3730xl DNA analyser (Thermo Fisher) and the results were analysed using the sequence alignment software from DNASTAR (Lasergen, Madison, WI, USA) to identify polymorphism relative to the Belém strain reference sequence from NCBI (EU401923).

Amino acid sequences were aligned by using ClustalW and the phylogenetic tree was reconstructed by the neighbour-joining (NJ) algorithm using the Jones-Taylor-Thorton (JTT) amino acid substitution model, as implemented in the MEGA v6 program. The reliability of the obtained tree was calculated by the bootstrap test based on 100 resamplings.

*Determination of chloroquine and mefloquine sensitivity -* We determined the sensitivity of*P. vivax*isolates towards chloroquine sulphate and mefloquine hydrochloride (Sigma-Aldrich), which were aliquoted in pre-dosed tubes (15 mg/tube). Chloroquine was dissolved in 3 mL of 100% ethanol and 7 mL of Roswell Park Memorial Institute (RPMI)-1640 medium (Gibco, Invitrogen Life Technologies, California, CA, USA) and mefloquine was dissolved in 10 mL of 100% methanol. From the stock solution, another solution was prepared for each drug at final concentrations of 600 ng/mL for chloroquine and 300 ng/mL for mefloquine, in a 3:1 mix (vol/vol) of RPMI-1640 medium and Waymouth Medium (Sigma-Aldrich)*.* Then, 100 µL of each dilution was added into all the wells in column 1 of the 96-well tissue culture plates (Falcon, Corning, NY, USA), and nine subsequent two-fold dilutions were added into the wells in columns 2 to 9. Wells in columns 10 to 12 were filled with 50 μL of complete culture medium (culture control wells). The concentration of each of the antimalarial drug was tested in quadruplicate.

Samples with parasitaemia ranging from 0.1 to 1% were used directly, whereas samples with parasitaemia higher than 1% were diluted with uninfected O-positive-group erythrocytes to obtain a final parasite density of 0.1 to 1%. Blood samples were washed twice with a solution of RPMI-1640 medium and then resuspended in RPMI-Waymouth (Sigma-Aldrich). Finally, 200 μL of this suspension was added into each well in the antimalarial pre-dosed plates at a 1.2% final haematocrit. The plates were incubated for 48 h at 37ºC in a CO_2_ incubator (5% CO_2_ in air) and then frozen and kept at -20ºC. Before enzyme linked immunosorbent assay (ELISA), the plates were subjected to three consecutive freeze-thaw cyclesin order to lyse the red blood cells.

*ELISA -* The success of the drug sensitivity assay and the appropriate volume of the haemolysed culture were previously determined for each clinical isolate by a preliminary LDH ELISA as a pre-test. To determine which dilution of the haemolysed culture had to be used in the Deli-test, four serial dilutions (1:50, 1:25, 1:12.5 and 1:6.25) of the culture control wells (no drug) of each isolate were tested in a preliminary LDH ELISA. The dilutions were selected based on the wells that displayed optical density (OD) readings ranging from 1 to 2.

ELISA plate (Nunc, Maxisorp, Denmark) wells were coated with 100 μL of monoclonal antibody (MAb) against *P. vivax* (11D) LDH at 1 μg/mL in phosphate-buffered saline (PBS) (pH 7.4). The plates were incubated overnight at 4ºC, washed with PBS containing 1% bovine serum albumin (BSA) (fraction V, Boehringer-Mannheim, Mannheim, Baden-Wurttemberg, Germany) (PBS-BSA) and then incubated with 300 μL of PBS-BSA for 4 h at room temperature (RT). The plates were maintained at 4°C until further use.

Subsequently, the appropriate volume of the haemolysed culture was transferred to the wells of the ELISA plate with PBS-BSA to a final volume of 100 μL, incubated for 1 h at 37ºC, and then washed with PBS-BSA. After the addition of 100 μL per well of a biotinylated MAb against pan-*Plasmodium* LDH (19G7), the plates were incubated for 1 h at 37ºC. After washing, a third incubation was done for 30 min at RT with 100 μL of a streptavidin horseradish peroxidase solution followed by a final washing step. The enzyme activity was revealed after 5 min of incubation at RT with 100 μL of tetramethylbenzidine (TMB). The reaction was stopped with 1 M of phosphoric acid and the absorbance was read at 450 nm in a spectrophotometer (Spectramax 250, Molecular Devices, San José, CA, USA).

The concentration-response data were analysed using non-linear regression function to determine the 50% inhibitory concentration of parasite growth (IC_50_), defined as the concentration of the drug required to inhibit 50% of the production of lactate dehydrogenase (LDH) as determined by OD values from sample test wells compared to those obtained from drug-free control wells. The IC_50_ threshold values for resistance to chloroquine and mefloquine were 100 nM and 30 nM, respectively, and these values were consistent with previously described results.^20^

*Statistical analysis* - The data was stored in the Fox-plus^®^ (Borland International Inc, Perrysburg, OH, USA) data bank software. GraphPad Instat and GraphPad Prism (GraphPad Software Inc, San Diego, CA, USA) statistical software programs were used for data analysis. Student’s *t* test was used to analyse the differences in IC_50_ mean values, and the chi-square test was applied to compare the prevalence of isolates with a resistance profile.

## RESULTS

*Characterisation of P. vivax CSP variants -* Among the 95 isolates analysed, alleles of five different sizes - 1135, 1108, 1081, 1054 and 1027 bp - were amplified with the AL60/AL61 primers and alleles of sizes 786, 759, 732, 705 and 678 bp were amplified with PVCSP1/PVCSP2. The most common allele size was 1135/786 bp, corresponding to the 20 repeat units observed in 44 isolates (46.3%). The allele sizes of 1108/759, 1081/732, 1054/705 and 1027/678 bp corresponding to the 19, 18, 17 and 16 repeat units were observed in 19 (20%), 26 (27.4%), 2 (2.1%) and 4 (4.2%) isolates, respectively. All of the analysed isolates presented only one type of fragment (single infection). In addition to these samples,*P*.*falciparum*specimens were also tested, but showed negative PCR results with AL60/AL61 or PVCSP1/PVCSP2 primers. Therefore, the 95 samples from individuals infected with*P*.*vivax,*amplified by PCR, were subjected to sequencing reactions to screen the possible nucleotide polymorphisms of the gene encoding the PvCSP.

**Fig. 1:**
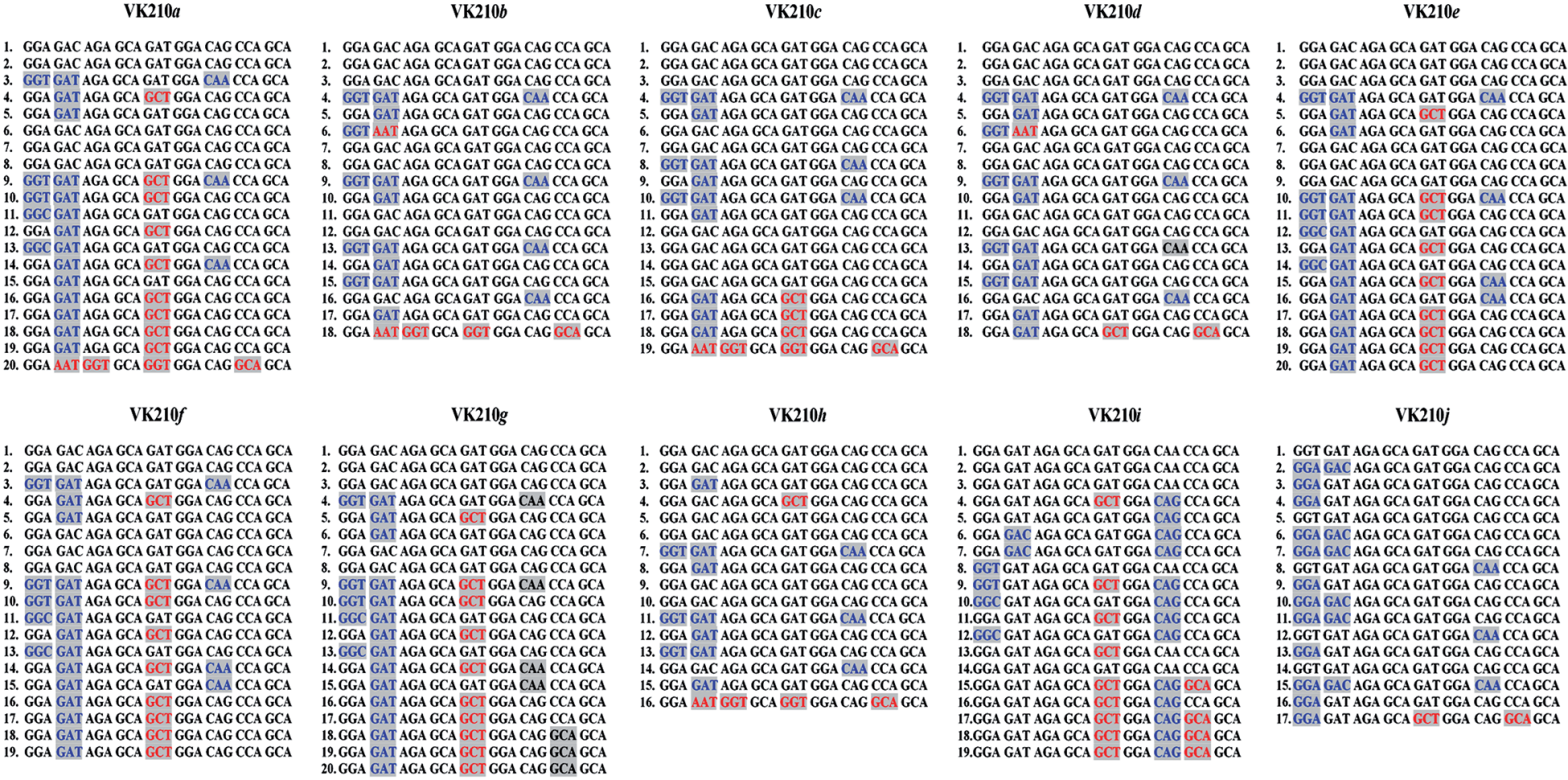
nucleotide sequence alignment for the central repetitive region (CRR) region of the *csp* gene found in *Plasmodium vivax* isolates with VK210 variant from Brazil. The numbers on the right represent the numbers of nonapeptides presented by the CRR. The letters in blue encode the same amino acid; letters in red encode different amino acid.

**Fig. 2:**
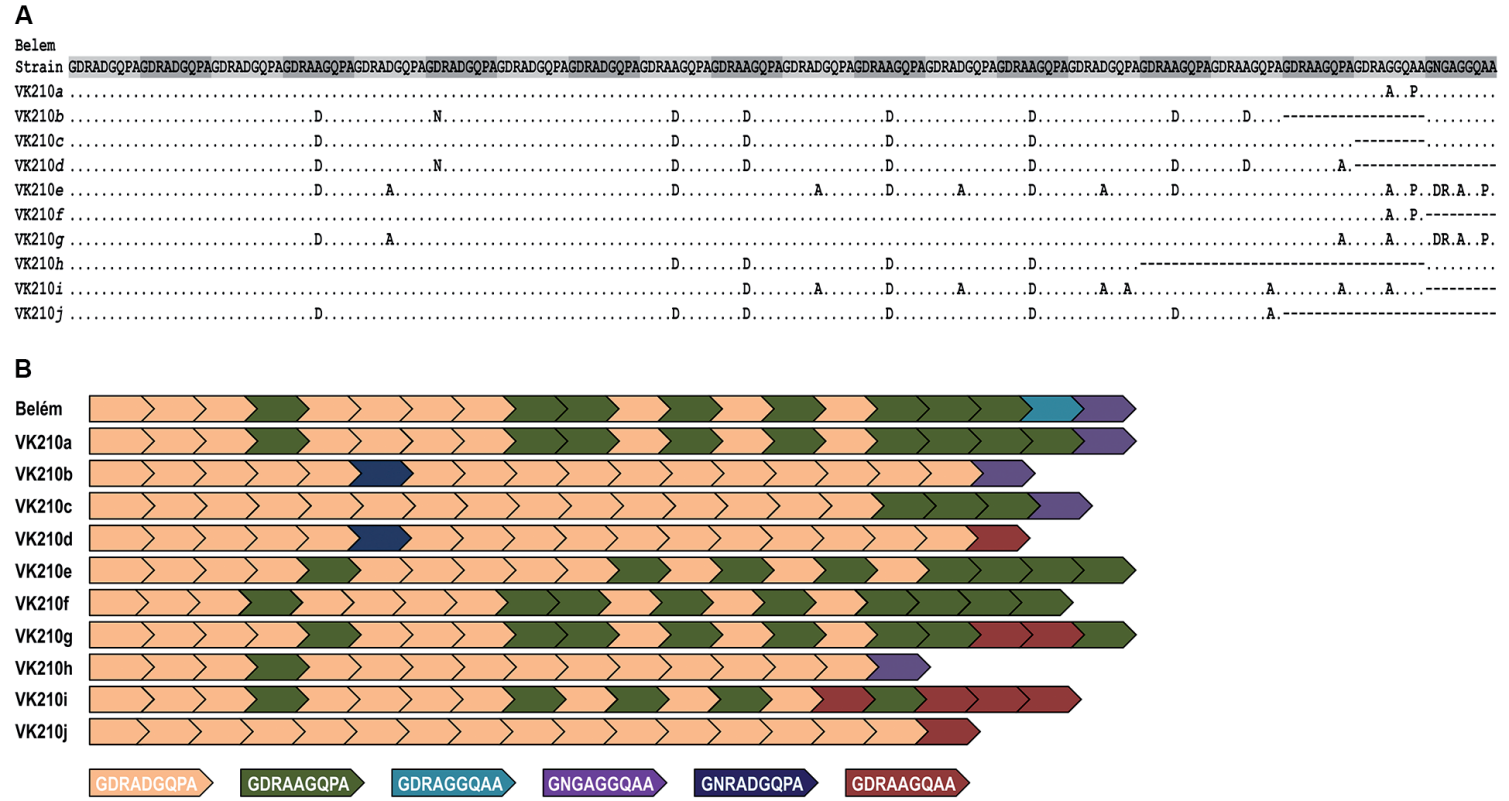
amino acid sequence alignment for the central repetitive region (CRR) peptide encoded by the *csp* gene in the *Plasmodium vivax* isolates from Brazil with the VK210 variant. A: the isolates obtained from individuals residing in Manaus (state of Amazonas) were aligned with the Belém reference strain (GenBank: EU401923). Dots represent identical residues and dashes represent deletions. The sequences highlighted in gray are the units of CRR; B: schematic representation of the CRR. Different colours represent each of the six nonapeptide repeats found in VK210 subtypes.

**Fig. 3:**
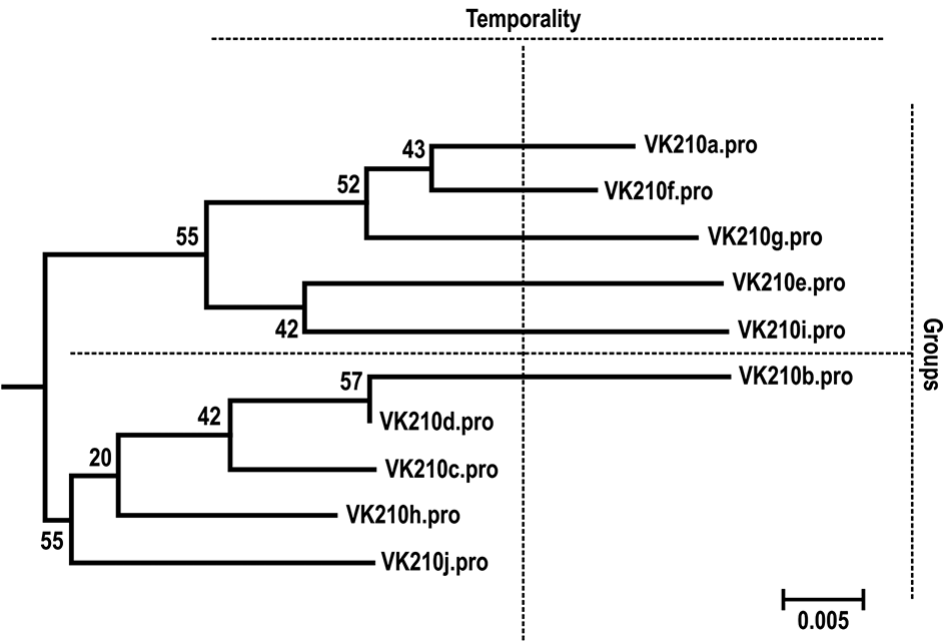
neighbour-joining tree of the *Plasmodium vivax* isolates obtained from individuals residing in Manaus (state of Amazonas) based on the nucleotide sequence of the circumsporozoite protein central repetitive region (CRR). The bootstrap values are shown on the branches and indicate the number of times out of 100 resamplings.

Using PCR-sequencing, we identified that all 95 samples from the isolates obtained in Manaus were of the VK210 variant. However, a great genetic diversity in CRR was observed, resulting in 10 different VK210 subtypes, named from VK210*a* to VK210*j* (Fig. 1)*.* These VK210 subtypes differed in numbers, varying from 16 to 20, and in the arrangements of five different nonapeptide sequences presented in the CRR: GDRADGQPA, GDRAAGQPA, GNRADGQPA, GDRAAGQAA and GNGAGGQAA (Fig. 2). Fig. 3 shows the phylogenetic relationship of the 10 VK210 subtypes from the isolated from Manaus based on the nucleotide sequence of the CRR.

The most frequent subtypes were the VK210*a* and VK210*b* found in 38 (40%) and 19 (20%) of the *P. vivax* isolates studied, respectively. The subtypes VK210*i*, VK210*g* and VK210*j* were poorly represented as they were present in only one (1%), two (2.1%) and two (2.1%) of the samples, respectively (Table).

**TABLE:** Frequency of VK210 subtypes in *Plasmodium vivax* isolates obtained from individuals living in Manaus, state of Amazonas, Brazilian Amazon

VK210 subtype	n	%
VK210*a*	38	40
VK210*b*	19	20
VK210*c*	11	11,6
VK210*d*	7	7,4
VK210*e*	4	4,2
VK210*f*	7	7,4
VK210*g*	2	2,1
VK210*h*	4	4,2
VK210*i*	1	1
VK210*j*	2	2,1
Total	95	100

n: number of isolates presenting the corresponding VK210 subtype.

**Fig. 4:**
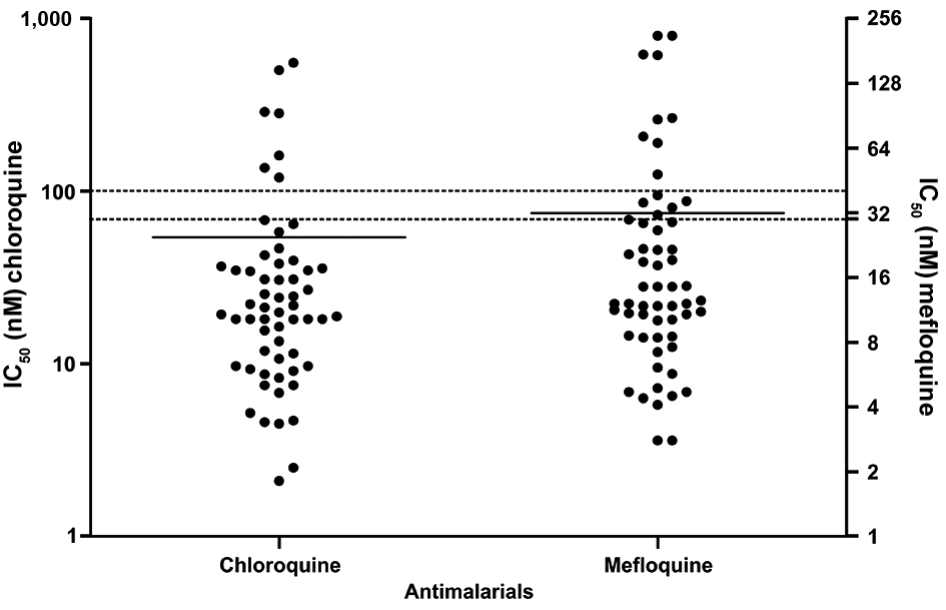
distribution of 50% inhibitory concentration (IC_50_) values in *Plasmodium vivax* isolates for chloroquine and mefloquine using Deli-test. The values correspond to individual IC_50_ values. Lines represent geometric mean. The dotted line represents the resistance threshold for chloroquine (100 nM) and for mefloquine (30 nM).

*Sensitivity profile of chloroquine and mefloquine resistance -* Overall, the IC_50_ values could be determined in most of the *P. vivax* isolates: 59/95 (62%). For chloroquine, the frequency of *P. vivax* isolates with IC_50_ above the threshold of 100 nM was 11.8% (7/59) and the geometric mean for the IC_50_ was 53.9 nM (59.3 ± 105.5 nM). For mefloquine, the frequency of isolates with a profile of resistance (IC_50_ > 30 nM) was 23.8% (14/59). The geometric mean IC_50_ for mefloquine was 31.8 nM (31.8 ± 48 nM) (Fig. 4). 

**Fig. 5:**
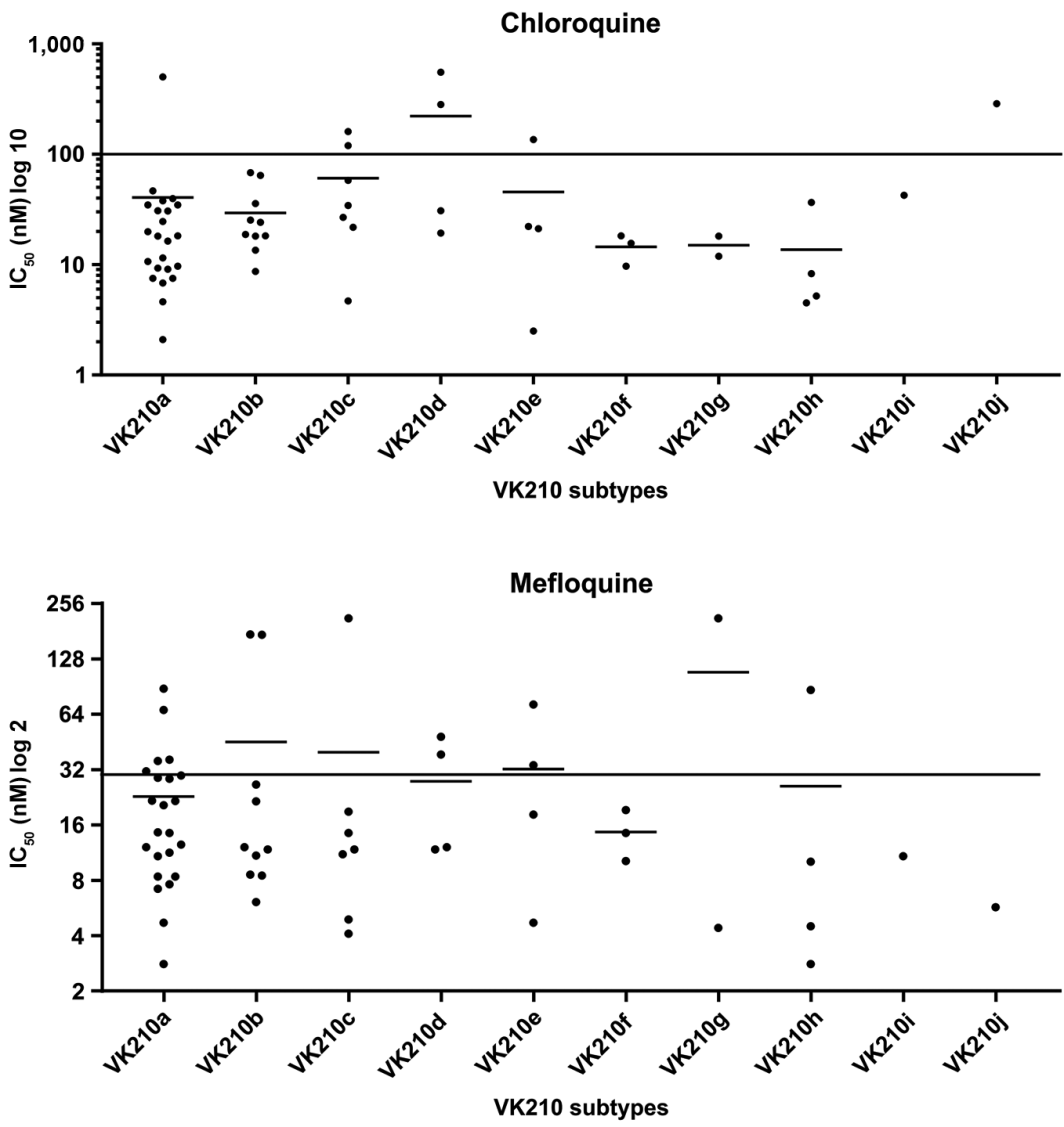
distribution of 50% inhibitory concentration (IC_50_) values in *Plasmodium vivax* isolates for chloroquine and mefloquine using Deli-test according to VK210 subtypes. The values correspond to individual IC_50_ values. Lines represent the geometric mean. The continuous line represents the resistance threshold for chloroquine (100 nM) and mefloquine (30 nM)

No difference was observed in the frequency of the resistant isolates and in the IC_50_ mean for chloroquine or mefloquine based on the VK210 subtypes (Fig. 5). Similarly, there was no observable difference in the frequency of resistant isolates and in the IC_50_ mean for chloroquine or mefloquine when the isolates were separated temporally or divided into two groups according to their phylogenetic relationship (Group A comprises VK210*a*, VK210*f*, VK210*g*, VK210*e* and VK210*i* and Group B comprises VK210*b*, VK210*d*, VK210*c*, VK210*h* and VK210*j*) (Fig. 3).

## DISCUSSION

In the present study, we identified the CSP variants in *P. vivax* isolates from individuals residing in Manaus and showed the difference in the sensitivity profiles of these specimens towards chloroquine or mefloquine. Among the 95 *P. vivax* isolates analysed, five different allele sizes were amplified by PCR, although the sequencing results showed that all the studied isolates were of the VK210 variant, with no occurrence of VK247 or *P. vivax*-like. Similarly, a study performed in a low endemicity area in the state of Acre, Amazon Basin of Brazil, also reported only the VK210 variant in the sympatric *P. vivax* isolates.^21^ These findings are consistent with results from previous studies reporting that the VK210 variant is the most frequent in malaria-endemic areas of the Brazilian Amazon.^12^ ^,^ ^13^ ^,^ ^22^ In fact, Machado and Póvoa^12^ suggested that VK210 is the best-adapted variant in Brazil as well as the world, as this variant has also been reported to be predominant in cases from Afghanistan, Iran, Azerbaijan, India, Thailand and Guiana.

However, we did not observe the VK247 variant in our study. This indicate that perhaps the VK247 subtype is not yet fully adapted in all Brazilian malaria-endemic areas, unlike earlier reports from endemic areas in Colombia where the frequency of the VK247 variant was seen to be higher.^23^ Alternatively, it might also be due to the differential susceptibility of *Anopheles* mosquitoes to infection with the *P. vivax* isolates^15^ as previous studies have reported differences in the infectivity of the anophelines to the variants, indicating that *Anopheles darlingi* were more susceptible to infection by VK210.^24^ We also cannot exclude the possibility that the lack of detection of the VK247 variant might be due to the low number of samples sequenced.

Individuals residing in malaria-endemic areas can be infected with genetically distinct parasite genotypes, which may result from either multiple infectious mosquito bites or bites from mosquitoes infected with multiple parasite genotypes. The complexity of the infections may also vary considerably based on the differences in the epidemiological scenarios. In low transmission areas, individuals may have infections with a single or a few parasite genotypes, while in high transmissions areas, individuals may have infections with more than 10 genotypes.^25^ In the present study, no mixed infection (VK210/VK247 or VK210/*P. vivax*-like) was observed in any of the analysed samples, which might reflect the low endemicity of the studied area as the frequency of mixed-clone infections has been correlated with the intensity of transmission. Competition of different strains for limited resources within a host during their life cycles may be important for survival, leading not only to higher transmissibility, but also an increase of the virulence and emergence of drug resistance.^26^ ^,^ ^27^

Insertions and deletions in CRR, resulting from either sexual recombination during meiosis or intrahelical strand-slippage events during mitotic DNA replication,^28^ can generate novel CSP variants. Although only the VK210 variant has been found in the studied area, high genetic diversity in CRR was observed, resulting in 10 different VK210 subtypes, with the VK210*a* and VK210*b* subtypes being the most predominant ones*.* These VK210 subtypes varied in numbers and in the arrangements of five different nonapeptide sequences presented in the CRR. The nonapeptide sequences GDRADGQPA and GDRAAGQPA have also been observed in the *P. vivax* isolates from Sri Lanka, Azerbaijan, South Korea, Iran, Brazil, China, Philippines, Solomon Islands and Gabon; the nonapeptide GNGAGGQAA was found in *P. vivax* isolates from Sri Lanka, South Korea, Iran, Brazil, China, Philippines and Solomon Islands while the nonapeptides GNRADGQPA and GDRAAGQAA were described only in isolates from Brazil.^21^ ^,^ ^29^

The infection due to these CSP variants seems to influence factors such as symptom severity, humoral response patterns, parasite burden and cytokine balance.^10^ ^,^ ^17^ However, the influence of these variants on drug response remains unclear. *P vivax* isolates that are resistant to antimalarial drugs have been reported in several countries, including Brazil.^20^ In a study conducted in Thailand, Kain et al.^17^ suggested that the response towards chloroquine varies depending on the type of *P. vivax* as the VK210 variant and mixed infection VK210/VK247 took longer to clear, while VK247 tended to have a shorter duration. Later, a study conducted by Machado et al.^16^ reported the correlation between the *P. vivax* variant and the response to chloroquine. Thus, we characterised the *P. vivax* CSP variants and subtypes in the isolates with different sensitivity profiles towards chloroquine and mefloquine, as determined using the colorimetric Deli test to evaluate whether the CSP variant can mark a *P. vivax* population with a distinct antimalarial resistance profile.

Overall, the IC_50_ values could be determined in 62% of *P. vivax* isolates collected in Manaus. *P. vivax* isolates showed a significant proportion of isolates with reduced sensitivity to chloroquine and mefloquine, 11.8 and 23.8%, respectively. The usefulness of the DELI test to generate results that can influence malaria control and public health policies has been demonstrated in a previous publication.^20^ It is important to note that the *in vivo* outcome depends on several factors that cannot be evaluated *in vitro*, including the level of innate and acquired immunity. However*, in vitro* assays act as a preliminary warning system indicating a trend as the *in vitro* resistance may be indicative of clinical resistance.^20^

Temporal variation in the habitat of the pathogen may directly or indirectly aid in the selection of the genetic diversity,^30^ and the genetic diversity of the *csp* gene has been associated with response to treatment as based on the infecting *P. vivax* CSP variant, there can be a difference in response towards chloroquine.^14^ ^,^ ^16^ In this study, we investigated if the sensitivity towards chloroquine and mefloquine was associated with the VK210 subtypes, separated temporally (older or more recent isolates) or phylogenetically (individual or separated by groups). We did not observe any difference in the frequency of the resistant isolates and in the IC_50_ mean for chloroquine or mefloquine, according to VK210 subtypes. Similarly, we also did not observe any difference in the frequency of the resistant isolates and in the IC_50_ mean for chloroquine or mefloquine when the isolates were grouped temporally or separated by group. The data presented here indicated that the VK210 subtypes does not mark a *P. vivax* population with different profiles of sensitivity to antimalarial drugs.

A limiting factor of our study and data is the small number of samples used to determine the IC_50_ and, consequently, the small number of isolates profiled for decreased sensitivity and/or antimalarial resistance, which could explain VK247 and *P. vivax*-like variants not being detected in the studied samples.

The data reported here indicated that the VK210 variant is the most frequent subtype in this malaria-endemic area of the Brazilian Amazon and that there is great genetic variability in CRR of the *P. vivax* circumsporozoite protein. However, VK210 subtypes might not be a suitable marker for the different sensitivity profile of the *P. vivax* populations towards antimalarial drugs.
